# Remote Effect of Lower Limb Acupuncture on Latent Myofascial Trigger Point of Upper Trapezius Muscle: A Pilot Study

**DOI:** 10.1155/2013/287184

**Published:** 2013-04-28

**Authors:** Kai-Hua Chen, Kuang-Yu Hsiao, Chu-Hsu Lin, Wen-Ming Chang, Hung-Chih Hsu, Wei-Chi Hsieh

**Affiliations:** ^1^Department of Physical Medical and Rehabilitation, Chang Gung Memorial Hospital, Chiayi, Chiayi County 613, Taiwan; ^2^School of Medicine, Chang Gung University, Taoyuan 333, Taiwan; ^3^Department of Emergency Medicine, Chang Gung Memorial Hospital, Chiayi, Chiayi County 613, Taiwan; ^4^Department of Nursing, Chang Gung University of Science and Technology, Chiayi, Chiayi County 613, Taiwan; ^5^Department of Physical Medical and Rehabilitation, Chang Gung Memorial Hospital, Yunlin, Yunlin County 638, Taiwan; ^6^Graduate Institute of Clinical Medical Science, College of Medicine, Chang Gung University, Taoyuan 333, Taiwan

## Abstract

*Objectives*. To demonstrate the use of acupuncture in the lower limbs to treat myofascial pain of the upper trapezius muscles via a remote effect. *Methods*. Five adults with latent myofascial trigger points (MTrPs) of bilateral upper trapezius muscles received acupuncture at Weizhong (UB40) and Yanglingquan (GB34) points in the lower limbs. Modified acupuncture was applied at these points on a randomly selected ipsilateral lower limb (experimental side) versus sham needling on the contralateral lower limb (control side) in each subject. Each subject received two treatments within a one-week interval. To evaluate the remote effect of acupuncture, the range of motion (ROM) upon bending the contralateral side of the cervical spine was assessed before and after each treatment. *Results*. There was significant improvement in cervical ROM after the second treatment (*P* = 0.03) in the experimental group, and the increased ROM on the modified acupuncture side was greater compared to the sham needling side (*P* = 0.036). *Conclusions*. A remote effect of acupuncture was demonstrated in this pilot study. Using modified acupuncture needling at remote acupuncture points in the ipsilateral lower limb, our treatments released tightness due to latent MTrPs of the upper trapezius muscle.

## 1. Introduction

Acupuncture is commonly used in traditional Chinese medicine for relief of myofascial pain syndrome [1]. The efficacy of acupuncture depends on various factors, including the site of needling, the intensity of acupuncture, and the mode of needle stimulation. The needling can be placed at either local or distant acupuncture points, and several studies have reported effective pain relief [2–4]. When acupuncture has an effect on a location far from the site of needle insertion (usually along the same channel), it is called a “remote effect” [3].

The “intensity of acupuncture” is related to factors including the number of sites needled per session, number and frequency of sessions, and depth of needle insertion. The mode of stimulation depends upon the method of manipulation, which includes needle twisting and additional electrical stimulation (electroacupuncture). However, the various options regarding intensity of acupuncture have not yet been standardized. 

The remote effect of acupuncture in patients with active myofascial trigger points (MTrPs) has recently been reported [5–7]. However, the remote effect of acupuncture on upper body MTrPs from lower limbs has not been investigated.

MTrPs are considered as localized hyperirritable spots in palpable taut bands of skeletal muscle fibers [8–22]. The characteristics of an MTrP [8, 9, 13, 14, 16–24] include the following: (1) a discriminate tender spot in a palpable taut band; (2) a consistent and characteristic referred pain pattern upon compression of an MTrP; (3) a local twitch response elicited by snapping palpation on MTrPs in some muscles or by needling of MTrPs in almost all cases; (4) restricted range of stretch (or motion of the involved joint); and (5) associated referred autonomic phenomena including vasoconstriction, coldness, sweating, pilomotor response, ptosis, and/or hypersecretion. MTrPs can be classified as active or latent [15, 17, 18]. An active MTrP is characterized by spontaneous pain or pain in response to movement, while a latent MTrP is a sensitive spot with pain or discomfort in response to compression, only. In other words, patients with latent MTrPs may only have functional changes, such as limitation of range of motion (ROM), but no painful sensation combined with the functional change. It has been suggested that the treatment of latent MTrPs in patients with musculoskeletal pain may not only decrease pain sensitivity and improve the motor function, but it also prevent their transformation into active MTrPs, thereby preventing the development of myofascial pain syndrome [25]. Coincidentally, increasing numbers of studies have shown that some MTrPs are actually the acupuncture points, the so-called “Ah-Shi” points [26–29].

The aim of this study was to investigate the remote effect of modified acupuncture in the lower limbs of adults with latent MTrPs of their upper trapezius muscles. We hypothesized that modified acupuncture could suppress the irritability of latent MTrPs of the upper trapezius muscle after remote acupuncture in the ipsilateral lower limb, which would lead to the improvement in cervical ROM.

## 2. Methods

### 2.1. General Design

This study was approved by our Institutional Review Board, and all subjects gave their written informed consent prior to participation in the study.

**Figure 1:**
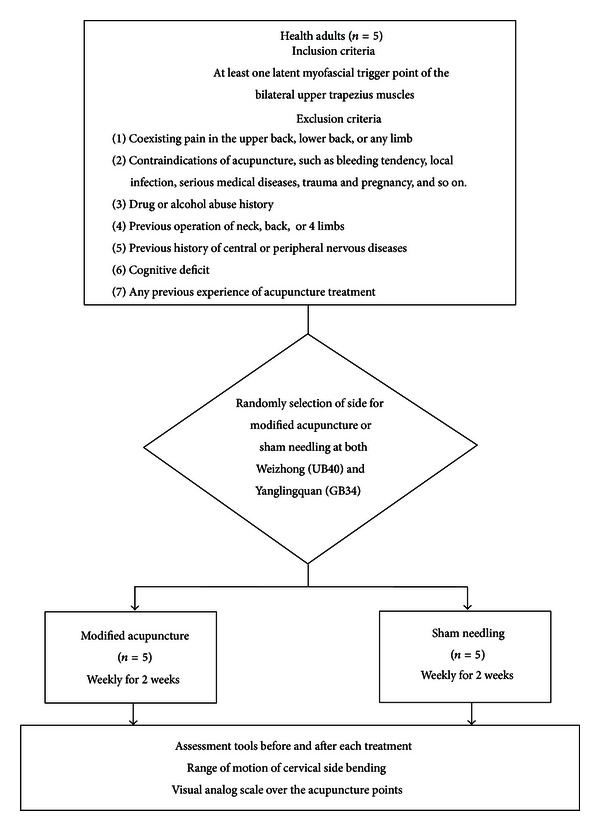
The flow diagram showing our study design.

Five adults with latent MTrPs of bilateral upper trapezius muscles received modified acupuncture therapy applied on a randomly selected ipsilateral lower limb (experimental side) versus sham needling on the contralateral lower limb (control side) in each subject. The acupuncture points in the ipsilateral lower limb selected for acupuncture therapy were Weizhong (BL40, on the posterior aspect of the knee, at the midpoint of the popliteal crease) and Yanglingquan (GB34, on the fibular aspect of the leg, in the depression anterior and distal to the head of the fibula) [30]. Each subject received one treatment on both lower limbs weekly for a total of 2 weeks. The ROM upon bending the contralateral side of the cervical spine was assessed before and after each treatment to evaluate for any remote effect. Pain intensity of the acupuncture site was also assessed using the visual analog scale (VAS) (Figure 1).

### 2.2. Subjects

All five adult subjects suffered from myofascial pain in their bilateral upper trapezius muscles for at least 3 months prior to enrollment in this study. Upon enrollment, pain over the upper trapezius muscle had diminished, but functional limitation in cervical ROM persisted. Besides, there was at least one latent MTrP of each upper trapezius muscle. The latent MTrPs were identified according to the following criteria: the presence of a sensitive, tender point in the palpable taut band within the upper trapezius muscles, pain induced only by external compression over the taut band with absence of spontaneous pain from this tender point. Exclusion criteria were coexisting pain in the lower back or any limb; contraindications for acupuncture such as bleeding tendency, local infection, severe medical illness, trauma, or pregnancy; history of drug or alcohol abuse which might interfere with the assessment of pain; a history of surgery on the neck, back, or any limb; a history of any neurologic disease involving either the central or peripheral nervous systems; cognitive deficits; and any history of prior acupuncture treatment.

A detailed explanation of the therapeutic and assessment procedure was given to each subject. All subjects were informed that they would receive one of two different acupuncture needling techniques for the relief of trapezius muscle tightness, but they were unaware as to which limb was chosen for the experimental side versus the control side. The decision regarding the choice of limb for the experimental acupuncture was determined by each subject who selected one of two envelopes containing either the letter “L” or “R”. The letter “L” indicated that the subject would receive modified acupuncture therapy on the left lower limb and “R” indicated the receipt of modified acupuncture therapy on the right lower limb.

### 2.3. Acupuncture Procedure

Each subject received acupuncture at both Weizhong (BL40) and Yanglingquan (GB34) acupuncture points using the modified acupuncture method on the randomly selected experimental side (modified acupuncture group) and sham needling on the contralateral limb (control group). Each subject received the same treatment to both lower limbs one week later. Each acupuncture point was treated for 3 minutes with the subject in the prone position with legs straightened in order to achieve maximal relaxation of the entire body and to avoid syncope during needling. One-inch, no. 30 gauge, disposable acupuncture needles were used. A licensed acupuncturist, who was blinded to the assessment, performed the procedure.

**Figure 2:**
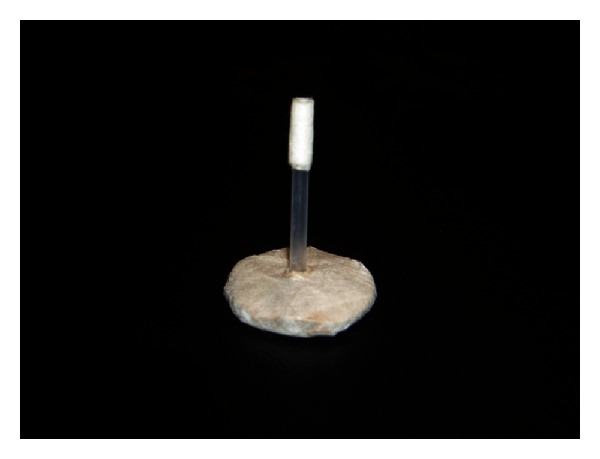
A small brass cylinder holds the acupuncture needle on the control side.

In the modified acupuncture group, the acupuncture needle was inserted into the muscular layer at a regular depth. The needle was then moved in-and-out for a distance of between 5–15 mm below the skin level and twisted clockwise and counterclockwise in multiple directions rapidly, as described by Chou et al. [6, 7], similar to the procedure for MTrP injection described by Hong [31]. As many as possible local twitch responses were elicited during the needle manipulation for 15 seconds, and then the acupuncture needle was fixed in place for 3 minutes. The “De-Qi” sensation was frequently elicited during this manner of needle manipulation. This sensation includes soreness, numbness, heaviness, and distension around the needle insertion region [32]. In the sham needling group, the acupuncture needle was inserted into the skin to a depth of 2 mm, and the needle was held in a small brass cylinder (Figure 2). No needle manipulations, such as moving in-and-out or clockwise or counterclockwise rotations, were performed. No “De-Qi” sensation or local twitch response was induced.

### 2.4. Assessments

The effect of acupuncture was determined by measuring the ROM upon bending the contralateral side of the cervical spine compared to the opposite side before and after the 1st and 2nd treatments. The VAS was also recorded in order to assess any painful sensations at the acupuncture sites before, and immediately after, each acupuncture treatment. All the assessment procedures were performed by one of the authors who was blinded to the group assignment of the participants.

### 2.5. Cervical ROM

The ROM upon bending the contralateral side of the cervical spine was measured with a large-scale goniometer to determine the degree of tightness of the upper trapezius muscles. During each measurement, the patient was instructed to sit on an adjustable chair with each hand placed on the front of each thigh. The height of the chair could be adjusted to keep the spinous process of the C7 vertebra at the same horizontal level as the center of the large-scale goniometer. The subject was then asked to bend his or her neck maximally to the right or left side for the least 5 seconds without any movement of the shoulders. Three consecutive measurements on each side were performed, and the average value was used for statistical analysis. If there was compensatory movement of shoulder and trunk during the measurement, the subject's position was rechecked, and he/she was given instructions on how to perform the measurement correctly.

### 2.6. VAS

As the participants with latent MTrPs had no pain at rest, the VAS was used only to monitor for any painful sensation at acupuncture site. The VAS score was recorded before, and immediately after, each acupuncture treatment. A card with an uncalibrated scale ranging from zero to ten on one side (with zero representing no pain and ten representing the worst imaginable pain) was shown to each participant. There was a corresponding 100 mm scale on the other side of the card. The participants subjectively estimated their pain level by moving the pointing device along the uncalibrated scale between zero and ten. The exact value of pain intensity was also read and recorded from the other side.

### 2.7. Statistical Analysis

The data from all measurements were collected before and after the 1st and 2nd acupuncture treatments. The degree of change in the values after the first treatment and before and after the second treatment was calculated using the following formula:

(1) Degree  of  change  (%) =100%   ×(post-treatment  value-pre-1st  treatment  value)(pre-1st  treatment  value)

A repeated-measures analysis of variance was used to compare the values before and after each treatment in each group. A paired *t*-test was used to compare the values between the modified acupuncture group and the sham needling group. *P* < 0.05 was considered to be statistically significant. All statistical analyses were performed using the Statistical Package for the Social Sciences Version 12.0 for Windows (SPSS Inc., Chicago, IL, USA).

## 3. Results

Five healthy adults (3 men, 2 women) with latent MTrPs of both upper trapezius muscles were recruited. Their ages ranged from 29 to 40 years with a mean age of 32.8 ± 5.3 years. All outcome assessments were expressed as mean ± standard deviation, as shown in Table 1.

Before the first acupuncture treatment, the ROM upon bending the contralateral side of the cervical spine measured 31.0 ± 7.7 degrees in the modified acupuncture group and 35.7 ± 8.9 degrees in the sham needling group. Because there was a significant difference in ROM between the two groups, data normalization, that is, the “degree of change,” was used for comparison. After the first modified acupuncture and sham treatment, there was no significant improvement in the ROM in either group (*P* > 0.05).

In the control group, there was no significant change in ROM after the second sham needling treatment (*P* > 0.05). In contrast, the ROM in the experimental group increased to 35.1 ± 9.0 degrees after the second modified acupuncture treatment. The degree of change was statistically significant, compared to the value before the second treatment (*P* = 0.030). The increased ROM on the modified acupuncture side was greater compared to the sham needling side (*P* = 0.036).

Because all participants in this study were adults with latent rather than active MTrP, the baseline VAS score for all subjects was zero. After the first acupuncture treatment, there was a transient increase in VAS score (0 versus 0.5 ± 0.3, *P* = 0.023) at the acupuncture site, which completely subsided before the second acupuncture treatment. There was no significant difference in VAS scores before and after the second acupuncture treatment (0 versus 0.7 ±  0.9, *P* = 0.171).

## 4. Discussion

In this study, participants with latent MTrPs were selected for the evaluation of a remote acupuncture effect using a modified technique. This remote effect was assessed by the degree of tightness of the upper trapezius muscle containing MTrP as determined by the degree of change in cervical ROM. We found that distal acupuncture, using a method similar to that used for myofascial pain injection, could relieve proximal muscular tightness and lead to an increase in cervical ROM. As our subjects with latent MTrP did not suffer from pain at baseline, the change in VAS score was measured over the acupuncture site only to ascertain whether any pain was induced by the acupuncture, itself. We found that transient pain developed only after the first acupuncture treatment, and, thus, adaptation to the pain after the first acupuncture occurred.

### 4.1. The Local and Remote Effects of Acupuncture

In previous reports in the literature, either local or distant acupuncture points were selected for pain relief in patients with myofascial pain. Ceccherelli et al. recruited patients with cervical myofascial pain [33]. The acupuncture points around the neck and shoulder girdle (such as Tianzhu (BL10), Fengchi (GB20), Dazhui (GV14), and Yamen (GV15)) and in the upper limbs (including Houxi (SI3), Waiguan (TH5), and Hegu (LI4)) were selected for eight sessions of stimulation. The authors found that the pain intensity evaluated by the McGill Pain Questionnaire was reduced at the end of treatment, and was sustained at 1 and 3 months later. This study supported the hypothesis that the cervical myofascial pain could be relieved by acupuncture at both local and distal acupuncture points. In the study by Irnich et al., a positive effect of distant acupuncture was also demonstrated [34]. Thirty-six patients with chronic myofascial neck pain were randomly assigned to receive one session of acupuncture at distant acupuncture points, dry needling in the MTrP of the neck and shoulder girdles, or sham laser at distant acupuncture points. Measurements of motion-related pain by VAS, cervical ROM in six directions, and change in verbal rating score were performed immediately after treatment. The selected acupuncture points were distributed in the posterior neck (GV20, GV14), trunk (KI 27, CV21, and CV22), upper limb (LU7, LI4), lower limbs (SI3, BL60), and ears (cervical spine, stellate ganglion). They found that the group with distant acupuncture treatment had lower scores by VAS, greater improvement in cervical ROM, and a greater decrease in verbal rating scores (*P* < 0.05 for all assessments). In a recent study by Chou et al. [6], distant acupuncture points in the upper limbs (TE5, LI11) were selected for the treatment of active MTrPs of the upper trapezius muscles. They also demonstrated a significant decrease in pain intensity and endplate noise amplitude in the MTrP region of the upper trapezius muscle.

In our study, the two acupuncture points selected for treatment, Weizhong (BL40) and Yanglingquan (GB34), were based on the traditional theory of “meridian” [35]. We found that modified acupuncture at these two acupuncture points in the lower limbs was effective in increasing cervical ROM. In the clinical practice in our country [35], Yanglingquan (GB34) is the principal acupoint for pain relief for myofascial pain syndrome. According to traditional theory, it is one of the eight meeting points where the “qi” of the tendon gathers. The tendon in classical literature indicates soft tissue (such as muscle, tendon, and fascia) in modern medicine. Weizhong (BL40) is the commonly recommended distant acupuncture point to relieve back pain [35].

### 4.2. The Issue of Acupuncture Application

The proper method of manipulation of the acupuncture needle is a key factor in achieving a positive effect from acupuncture. Ceccherelli et al. compared the effects of superficial acupuncture with those of deep acupuncture at both acupuncture points and trigger points in patients with lumbar myofascial pain [36]. In the superficial acupuncture group, the needle was introduced into the skin to a depth of 2 mm, whereas in the deep acupuncture group, the needle was inserted into the muscular layer to a depth of 1.5 cm into either the muscle or the trigger points. The same needle stimulation was performed in both groups (stimulation for 1 minute after insertion and for 20 seconds every 5 minutes at 5, 10, and 15 minutes); the frequency of alternative right and left rotations of the needles was 2 Hz. They found that the intensity of pain was reduced in both groups immediately after 8 sessions of treatments, but deep acupuncture was significantly more effective than superficial acupuncture therapy 3 months after the treatments. In that study, they concluded that superficial acupuncture was not really a “sham” therapy but a less effective type of acupuncture. This finding supports the effectiveness of superficial dry needling therapy [37–39]. In our study, we used superficial needling into the skin (2 mm in depth) in the control group. No movement (in-and-out) or clockwise-counterclockwise manipulation was performed. No “De-Qi” sensation or local twitch response was induced. The acupuncture needle remained fixed in place for 3 minutes. A painful sensation was only noticed when the acupuncture needle was inserted into the skin. No change in cervical ROM was observed after this treatment in our study. 

In a case reported by Chou et al. [5], acupuncture manipulation (similar to Hong's technique for MTrP injection) at distant acupuncture points was found to relieve proximal myofascial neck pain in a patient with fibromyalgia. The distant acupuncture points used by Chou et al. included acupuncture points in the back (SI11), upper limbs (GB21, TE14), and lower limbs (GB34, SP6). They later found that a remote influence exerted by the same needle manipulation at acupuncture points in the upper limb (Wai-huan, Qu-chi) was also effective for patients with active MTrP in the upper trapezius muscle [6]. In our study, we obtained similar findings by twisting the needle clockwise and counterclockwise alternatively during multiple insertions of the acupuncture needle (Chou's method) on distant acupuncture points in the lower limb and obtained similar findings. It is important to emphasize that both the “De-Qi” sensation and local twitch response were induced during needle manipulation in order to obtain a good result. Cervical ROM was improved after only two treatment sessions. Thus, the method of needle manipulation (and not the depth of needle) for eliciting local twitch response was the key factor in achieving a positive effect of acupuncture in our study. In addition, we selected the same acupuncture points (rather than a nonacupuncture point) for needling in the experimental versus control groups. As all points selected for needling were acupuncture points, we cannot exclude an “add-on” effect at the acupuncture points in this study.

### 4.3. The Method of Placebo Acupuncture

Several methods have been suggested for placebo acupuncture, including (1) sham acupuncture, (2) minimal acupuncture, (3) mock acupuncture, and (4) mock transcutaneous electrical nerve stimulation (TENS) [40]. Sham acupuncture has been described as insertion of the needle at some distance away from the acupuncture point with the same method of manipulation and stimulation as that inserted at the acupuncture point. It is the most commonly used placebo method. However, recent studies have shown that analgesic effects may also be produced through the activation of diffuse noxious inhibitory control [41]. Minimal acupuncture is performed by positioning the needles outside the acupuncture point and inserting the needles very superficially (1-2 mm) without any manipulation. This procedure can minimize the acupuncture effect. However, this very light stimulation may produce pain relief in some patients who are very responsive to acupuncture stimulation (called “strong responders”). Mock acupuncture is performed with a small brass cylinder with a blind end, which is fixed to the patients' skin with plaster. The needle is inserted inside this cylinder but the tip of needle does not contact the skin. The lack of needling sensation is explained to the patient as the result of superficial anesthesia. Similar to mock acupuncture, mock TENS is performed by placing the TENS electrodes on the target skin with only light or sounds emitted from the machine but with the current output switched off. The lack of stimulation is explained as “subliminal stimulation” to the patient. In our present study, the method of acupuncture in the control group was somewhat similar to the minimal and mock acupuncture methods. A small brass cylinder with two open ends was held by the acupuncturist on participant's skin. The length of the cylinder was just 2 mm shorter than the length of the acupuncture needle. Thus, we ensured that the depth of the acupuncture needle was only 2 mm below the skin, and, therefore, minimal acupuncture was given. No positive remote effects were found when this method was used in our control group. In addition, we selected the same acupuncture points (rather than a nonacupuncture point) for needling in the experimental and control groups. This design minimized bias between the acupuncture points and nonacupuncture points and also minimized bias among different acupuncture points. The only differences between the experimental and control groups were the depth and the method of needling.

### 4.4. Current Theory of the Mechanism of the Acupuncture Effect

Diffuse noxious inhibitory control has been used to explain the efficacy of acupuncture [41]. The diffuse noxious inhibitory control model asserts that an analgesic effect can be obtained by noxious stimulation to any part of the body, and the magnitude of the analgesic effect is also related to the magnitude of the noxious stimulus. The other explanation for pain control of MTrPs by acupuncture is secondary to hyperstimulation analgesia [26]. According to this theory, pain can be relieved by hyperstimulation of the pain fibers produced by dry needling or acupuncture. The pathway of hyperstimulation analgesia is probably also via the diffuse noxious inhibitory control. The moderate-to-intense sensory input of hyperstimulation analgesia can be applied locally or at a distance from the painful site. Recent data also supports the hypothesis that analgesia provided by local acupuncture occurs through the activation of large afferent fibers, whereas analgesia induced by distant acupuncture is mediated by the excitation of small afferent fibers if the stimulation from the distant acupuncture is strong enough [2].

### 4.5. Study Limitations

Our study had several limitations including the small sample size (only five subjects were recruited) and low frequency of treatments (only two acupuncture treatments were given within a 1-week interval). Nevertheless, statistically significant improvement in cervical ROM was found in the experimental group compared to the controls. It is hoped that the results of this pilot study may stimulate further research on the effectiveness of remote acupuncture, in patients with active MTrPs in upper trapezius muscles.

## 5. Conclusion

We found that the distal acupuncture by a method similar to MTrP injection could relieve proximal muscular tightness and lead to improvement in cervical ROM in individuals with latent MTrP. We concluded that a remote effect on proximal latent MTrPs in the upper trapezius muscle was created by modified acupuncture at distant acupuncture points in the lower limb.

## Figures and Tables

**Table 1:** The range of motion upon bending the contralateral side of the cervical spine in two groups before and immediately after acupuncture treatments.

Group	Before the 1st treatment	After the 1st treatment		Before the 2nd treatment		After the 2nd treatment		*P* value^†^			
	Pre-1st (degree)	Post-1st (degree)	DOC* (%)	Pre-2nd (degree)	DOC*(%)	Post-2nd (degree)	DOC*(%)	Pre-1st versus Post-1st	Pre-1st versus Pre-2nd	Pre-1st versus Post-2nd	Pre-2nd versus Post-2nd
Modified acupuncture	31.0 ± 7.7	32.8 ± 10.4	4.5 ± 10.6	30.1 ± 7.5	−1.9 ± 14.7	35.1 ± 9.0	14.7 ± 17.2	0.308	0.727	0.170	0.030^§^
Sham needling	35.7 ± 8.9	34.9 ± 3.8	1.1 ± 18.8	33.4 ± 7.2	−3.4 ± 24.5	34.6 ± 8.0	−1.6 ± 17.1	0.777	0.588	0.714	0.452
*P* value^‡^	0.021^§^		0.784		0.870		0.036^§^				

Pre-1st: the value before the first treatment; post-1st: the value after the first treatment; pre-2nd: the value before the second treatment; post-2nd: the value after the second treatment; and DOC: degree of change.

Values are mean ± standard deviation or *P* value.

*Degree of change was calculated by 100%  × (post-treatment value − pre-1st treatment value)/(pre-1st treatment value).

^†^Repeated measured ANOVA was used to compare the values within the same group.

^‡^Paired *t*-test was used to compare the values between two groups.

^§^ *P* < 0.05.
